# Noble Metal-Doped Perovskite–GO Hybrids as Efficient Electrocatalysts for Alkaline Water Electrolysis

**DOI:** 10.3390/nano16020107

**Published:** 2026-01-14

**Authors:** Bogdan-Ovidiu Taranu, Paula Svera, Doru Buzatu, Maria Poienar, Paula Sfirloaga

**Affiliations:** 1 National Institute for Research and Development in Electrochemistry and Condensed Matter Timisoara, Dr. Aurel Paunescu Podeanu Str. No. 144, 300569 Timisoara, Romania; b.taranu84@gmail.com (B.-O.T.); paulasvera@gmail.com (P.S.); buzatu_doru@yahoo.com (D.B.); 2 ICAM-Institute for Advanced Environmental Research, West University of Timișoara, Bd. V. Pârvan No. 4, 300223 Timisoara, Romania; maria.poienar@e-uvt.ro

**Keywords:** perovskite, graphene oxide, water splitting, electrocatalyst, hydrogen evolution reaction

## Abstract

Water electrolysis using electricity generated from renewable sources is a promising approach for producing green hydrogen. However, this process requires the development of electrocatalysts that are not only highly active and durable but also low-cost. Considerable efforts are being directed toward discovering and optimizing such materials, and this study contributes to the ongoing research in this area. In this work, three novel LaMnO_3_ perovskite–graphene oxide hybrids—namely LaMnO_3_/GO, Ag-doped LaMnO_3_/GO, and Pd-doped LaMnO_3_/GO—were synthesized and investigated for their electrocatalytic activity in water electrolysis under strongly alkaline conditions. To the best of our knowledge, these hybrid materials have not been previously reported in the context of electrocatalytic water splitting. Among the electrodes fabricated and tested for the hydrogen evolution reaction (HER), the one based on a catalyst ink containing Pd-doped LaMnO_3_/GO mixed with carbon black showed the best performance, achieving a low overpotential of 0.385 V at a current density of −10 mA/cm^2^. It also demonstrated good stability in the alkaline electrolyte and exhibited a Tafel slope of 0.34 V. These findings highlight the potential of the studied materials as effective and previously unreported electrocatalysts for water splitting.

## 1. Introduction

In today’s world, fossil fuel reserves are dwindling, the environment is polluted, temperatures are rising, there is overcrowding, and a continuous increase in energy demand. These issues have led to political tensions, as well as a global energy and economic crisis [1]. The scientific community is making substantial efforts to find solutions to these urgent matters by focusing especially on their energy-related aspects [2,3]. The direction in which the research is advancing is towards finding alternatives to using fossil fuels as the main energy source that are also environmentally friendly [4]. Within this context, hydrogen has been singled out in recent decades as a promising energy carrier, having the potential to bring about a new energy infrastructure that is no longer based on the burning of fossil fuels [5]. Among other desirable properties, hydrogen has high gravimetric energy density and combusts without polluting the environment [6]. When generated using only renewable energy sources, it is known as green hydrogen, and water electrolysis is the usual method employed to obtain it [7]. This approach was initially reported in 1789 and involves two half-cell reactions, namely the hydrogen evolution reaction (HER) unfolding at the cathode and the oxygen evolution reaction (OER) occurring at the anode [8]. The overall reaction is presented in Equation (1), while Equations (2) and (3) show HER and OER in basic electrolyte solutions [9].

(1) 2H_2_O → 2H_2_ + O_2_

(2) 4OH^−^ → 2H_2_O + O_2_ + 4e^−^

(3) 4H_2_O + 4e^−^ → 2H_2_ + 4OH^−^

The heavy reliance on water and its environmental friendliness make water electrolysis a very advantageous process with the potential to provide a sustainable alternative to the fossil fuel-dependent energy infrastructure. However, while the water splitting reaction can unfold at RT and atmospheric pressure, electrocatalysts are required to efficiently lower the H_2_ and O_2_ overpotential values [10]. This places major focus on the synthesis of highly active and long-term stable electrocatalytic materials. Currently, precious metals and precious metal-based materials such as Pt for HER and IrO_2_ and RuO_2_ for OER are regarded as the most active electrocatalysts, ensuring low overpotentials even at high current density values [11]. However, noble metals substantially increase the cost of the water electrolysis process, which is already relatively high due to the high electric power consumption [12]. While the latter issue can be addressed using electricity generated using renewable sources (wind power, tidal power, or solar energy), finding a suitable replacement for the noble catalysts has become a daunting task [13]. Furthermore, noble metals are scarce, which is another factor why they cannot be used in large-scale water splitting applications [14]. More affordable and earth-abundant materials are required for electrocatalyst synthesis. Catalysts with low noble metal content, chalcogenides containing transition metals, transition metal oxides, carbides, sulfides, phosphides, nitrides, alkoxides, hydroxides, carbon-supported single-atom catalysts, and other metal-free compounds have been studied regarding their water splitting electrocatalytic properties [9,15,16,17,18]. Oxide-based catalysts, such as ABO_3_ perovskite oxides, are attractive due to their specific physical–chemical and electronic properties. The flexibility of perovskite oxides allows their substitution at the A and B sites with most known elements, often with a rare-earth metal at the A site and a transition metal at the B site [19]. Furthermore, perovskites can also be doped and used as constituents of hybrid materials. For example, Sfirloaga et al. [20] reported the synthesis of two hybrid structures, namely polyvinylpyrrolidone (PVP) functionalized with Ag-doped LaMnO_3_ and Pd-doped LaMnO_3_, which were subsequently shown to exhibit OER activity in an alkaline environment.

The present study continues the search for materials based on doped ABO_3_ perovskites with potential application in the field of water electrolysis. Specifically, three LaMnO_3_ perovskite–graphene oxide hybrids were synthesized and evaluated in terms of their HER electrocatalytic activity in a strong alkaline electrolyte solution. These are undoped LaMnO_3_/GO (LMG), Ag-doped LaMnO_3_/GO (LMG-Ag), and Pd-doped LaMnO_3_/GO (LMG-Pd). To the best of the authors’ knowledge, no previously reported studies have investigated LaMnO_3_/GO hybrids—either doped or undoped—for electrochemical water splitting applications. The results obtained herein, using electrodes modified with catalyst inks containing the hybrid electrocatalysts drop-casted on the surface of glassy carbon substrates, show that the electrode modified with LMG-Pd, in combination with carbon black, exhibits the lowest HER overpotential value.

## 2. Materials and Methods

### 2.1. Materials and Reagents

**Scheme 1:**
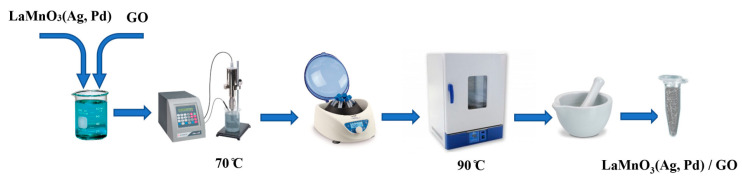
The stages of the functionalization procedure.

Perovskite-type materials based on LaMnO_3_, both undoped and doped with noble metals (Pd and Ag), were functionalized with graphene oxide (GO) using an ultrasound-assisted method involving a sonotrode immersed in an aqueous medium. The preparation method of pristine LaMnO_3_ and 1% Ag- or Pd-doped LaMnO_3_ perovskites has been previously reported in detail in our published work [21]. Graphene oxide was employed as an aqueous dispersion with a concentration of 4 mL/mL, and the weight ratio between the perovskite material and GO was maintained at 10:1 for all samples. For each sample (LMG, LMG-Pd, and LMG-Ag), 0.1 g of perovskite powder was mixed with 2.5 mL of GO dispersion (corresponding to approximately 0.01 g GO) in a Berzelius beaker and further diluted with ultrapure distilled water. The mixtures were subjected to ultrasonic treatment for 30 min using a Sonics Vibra Cell ultrasonic processor (Artisan Technology Group, Champaign, IL, USA) equipped with a titanium probe immersed directly in the reaction medium. The ultrasonic parameters were as follows: total time = 30 min, pulse mode = 10 s ON/5 s OFF, controlled temperature = 70 °C, and amplitude = 89%. After sonication, the samples underwent separation and drying steps. Specifically, they were centrifuged at 6000 rpm for 10 min and then dried in an oven at 90 °C for approximately 2 h. This procedure enabled the effective anchoring of graphene oxide onto the perovskite surfaces under controlled thermal and ultrasonic conditions, leading to the formation of hybrid nanomaterials with potential applications as electrocatalysts and advanced functional materials. Scheme 1 shows the stages of the functionalization procedure.

For the electrochemical experiments, glassy carbon (GC) pellets (8 mm in diameter) were acquired from Andreescu Labor & Soft SRL (Bucharest, Romania). Carbon Black-Vulcan XC 72 was procured from Sigma Aldrich (Saint Louis, MO, USA), and Nafion^®^ 117 solution was purchased from Fuel Cell Store (Bryan, TX, USA). KOH and KCl were obtained from Merck (Darmstadt, Germany), while absolute ethanol (99.5%) was acquired from ChimReactiv (Bucharest, Romania). All reagents were used as received, and double-distilled water was utilized to prepare all electrolyte solutions.

### 2.2. Electrode Development Method

Catalyst inks were obtained by dispersing the powder hybrid materials in ethanol. The perovskite hybrids were added alone or in a mixture with carbon black. Homogeneous dispersions resulted from applying an ultrasonication treatment for 30 min. In the next stage of the electrode preparation process, a volume of 10 µL was taken from each electrocatalyst-containing suspension and drop-casted onto the surface of the GC pellets. The modified pellets were dried at 40 °C until solvent evaporation, resulting in the electrodes specified in Table 1.

This method for obtaining modified electrodes has been previously reported in studies aimed at evaluating the water electrolysis properties of electrocatalysts [22,23]. The GC pellets served as conductive substrates, carbon black is known to improve charge transport, and Nafion acts as a binder by preventing the detachment of the deposited particles from the substrate [24,25].

### 2.3. Electrochemical Experiments

All water electrolysis experiments were performed with a potentiostat, type Voltalab, model PGZ 402, purchased from Radiometer Analytical (Lyon, France). Three electrodes were connected to this apparatus and immersed in the electrolyte solution added to a standard electrolysis cell. Each modified electrode served as the working electrode. Furthermore, an unmodified GC pellet and an electrode modified using a carbon black-based catalyst ink without any electrocatalyst were also prepared and used as working electrodes. The pellets were studied after being inserted into a polyamide support, which limited the geometrical surface in contact with the electrolyte solution to 0.28 cm^2^. The auxiliary electrode was a Pt plate with a geometric surface of 0.8 cm^2^, and an Ag/AgCl (sat. KCl) electrode was used as reference. The strongly alkaline electrolyte solution was 1 M KOH. It was deaerated by bubbling with high-purity nitrogen before each HER test. The recorded cathodic polarization curves were iR-corrected and were obtained at a scan rate v = 5 mV/s. Well-known equations were used to represent the electrochemical potential (E) values in terms of the reversible hydrogen electrode (RHE) and to determine the HER overpotential (η_HER_), the Tafel slope, the capacitive current density (i_dl_), the roughness factor (R_f_), and the electrochemically active surface area (ECSA) for the tested samples [23].

### 2.4. Physical-Chemical Analyses

Powder X-ray diffraction (XRD) measurements were performed using an X’Pert Pro diffractometer (PANalytical, Almelo, The Netherlands) equipped with a Cu Kα radiation source (λ = 1.5406 Å). Data were collected over a 2θ range of 10° to 80°, using continuous scan mode with a step size of 0.02° and a scan rate of 2°/min. The resulting diffraction patterns were analyzed using the HighScore Plus software Version: 2.2b (2.2.2) (PANalytical). Peak positions and intensities were further used to assess the phase purity and crystallinity of the hybrid materials.

Atomic force microscopy (AFM) images were acquired using a MultiView 2000 scanner (Nanonics Imaging Ltd., Jerusalem, Israel), in intermittent mode and with a tip radius of 20 nm.

Raman analysis was performed on the most electrocatalytically active electrode identified during HER experiments, both before and after the electrochemical stability test. The spectra were recorded using a MultiView-2000 system (Nanonics Imaging Ltd., Jerusalem, Israel) with a Shamrock 500i spectrograph (Andor, Essex, UK). The excitation wavelength was 514 nm.

Nitrogen adsorption–desorption measurements were carried out using a Quantachrome Nova 1200e analyzer (Quantachrome Instruments, Boynton Beach, FL, USA) at 77 K. Prior to analysis, the samples were degassed under vacuum at 40 °C for 12 h. The specific surface area was determined using the Brunauer–Emmett–Teller (BET) method, while the pore size distribution was calculated using the Barrett–Joyner–Halenda (BJH) method. The total pore volume was estimated from the amount of nitrogen adsorbed at the highest relative pressure on the adsorption branch of the isotherm.

## 3. Results and Discussion

### 3.1. XRD and AFM Characterization of the Hybrid Materials

**Figure 1:**
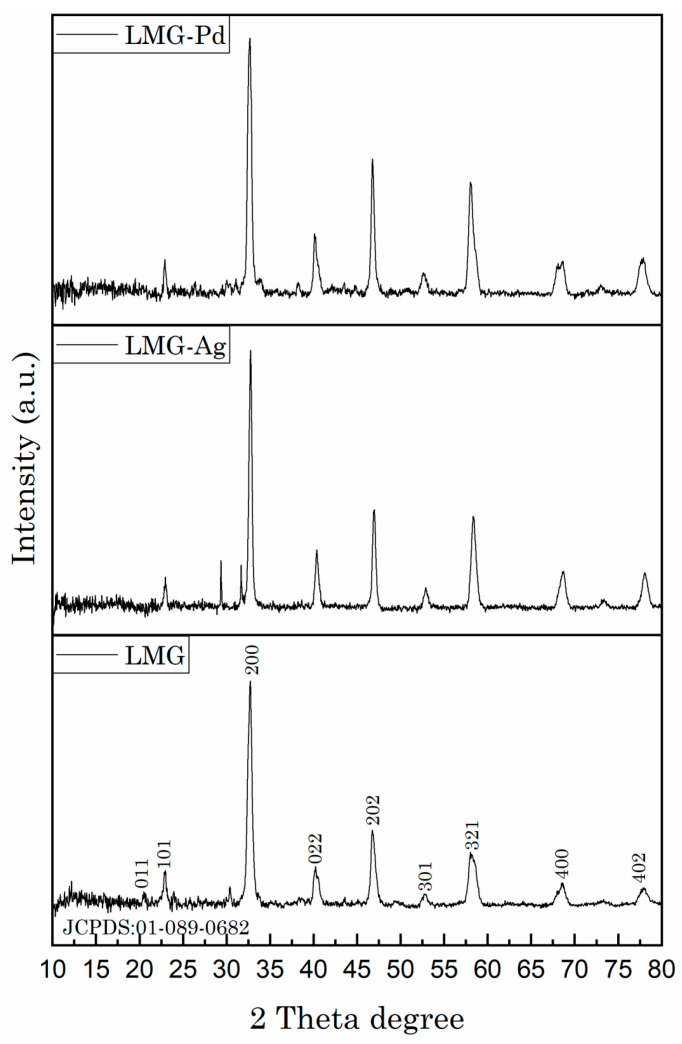
XRD patterns for the LMG, LMG-Ag, and LMG-Pd materials.

X-ray diffraction (XRD) analysis (Figure 1) was performed to comprehensively investigate the crystalline structure and phase composition of the synthesized hybrid materials. The diffraction patterns of the pristine perovskite (LMG) and the Ag- and Pd-doped samples (LMG-Ag and LMG-Pd) exhibit well-defined and sharp peaks at approximately 31°, 37°, 45°, 55°, 58°, 66°, and 74°. These reflections correspond closely to the characteristic planes of the perovskite crystal lattice, indicating that the fundamental perovskite structure is preserved despite the doping or functionalization processes [26]. Some small additional peaks are observed in the X-ray diffraction patterns, which originate from secondary phases introduced during the synthesis (e.g., NaOH, nitrates, and related byproducts) that were not completely removed during the washing process.

The preservation of the perovskite phase after doping suggests that the incorporation of Ag and Pd ions into the lattice does not significantly disrupt the crystal framework. This observation is crucial, as it confirms the structural stability of the perovskite matrix and indicates successful doping without phase separation.

The characteristic reflections can be readily indexed to the (011), (101), (200), (022), (202), (301), (321), (400), and (402) planes, according to JCPDS card No. 01-089-0682, corresponding to an orthorhombic structure with space group Pnma (no. 62), and unit cell parameters: a = 5.72 Å, b = 7.67 Å, c = 5.53 Å, with α = β = γ = 90°.

Furthermore, subtle variations in peak intensities and slight shifts in peak positions among the samples suggest minor lattice distortions or changes in crystallinity. These changes can be attributed to the difference in ionic radii between the dopant ions (Ag^+^ and Pd^2+^) and the host lattice cations, which may induce local strain or defects within the crystal lattice [27]. Such modifications can influence the electrocatalytic properties of the material, highlighting the importance of controlled doping.

Regarding the graphene oxide (GO) functionalization, the typical broad and weak diffraction peak associated with the (001) plane of GO around 10–12° is not prominently observed, which could be due to the low GO content within the composite. The absence of a clear GO peak suggests a good dispersion and interaction between GO sheets and the perovskite particles, which is beneficial for enhancing the hybrid material’s surface area and electronic or catalytic properties [28].

In summary, the XRD analysis confirms that the perovskite structure remains largely intact following doping with Ag and Pd and functionalization with graphene oxide. The structural integrity, combined with the absence of phase segregation, supports the successful synthesis of hybrid materials with potentially enhanced functional properties.

The AFM investigation was performed on samples obtained using the same procedures as for the GC_LMG_, GC_LMG-Ag_, and GC_LMG-Pd2_ electrodes. The recorded AFM images are presented in Figures S1–S3 from the Supplementary Material file. The values of the AFM parameters obtained from the AFM images, namely the average roughness (Sa), mean square root roughness (Sq), maximum peak height (Sp), maximum valley depth (Sv), maximum peak-to-valley height (Sy), surface kurtosis (Sku), and surface skewness (Ssk), are shown in Table S1. The highest roughness values were found for the GC_LMG-Pd2_ sample (Sa = 184.2 nm and Sq = 233.6 nm), followed by the values found for the GC_LMG-Ag_ sample (Sa = 62.82 nm and Sq = 77.77 nm) and for the GC_LMG_ sample (Sa = 61.36 nm and Sq = 75.01 nm), respectively. In the case of the GC_LMG-Ag_ and GC_LMG_ samples, it is not just the values determined for the Sa and Sq parameters that were found to be similar, but also those for Sp, Sv, and Sy. However, GC_LMG-Ag_ features more valleys, as suggested by the Ssk value and the slightly higher Sv value, indicative of a more porous surface. Morphologically, the surface of the GC_LMG_ sample displays more clusters, whereas rounder and bigger shapes are observed on the surface of GC_LMG-Ag_, which may be a consequence of the Ag-doping. The GC_LMG-Pd2_ sample stands out in terms of both roughness and morphology. Apart from the higher Sa and Sq values, this is the only sample for which a positive Ssk value was found, indicating that its profile was skewed downward relative to the mean plane [29]. Lastly, Sku values < 3 were determined for all the samples, which are characteristic of surfaces with relatively few high peaks and low valleys [30].

### 3.2. BET Analysis

**Figure 2:**
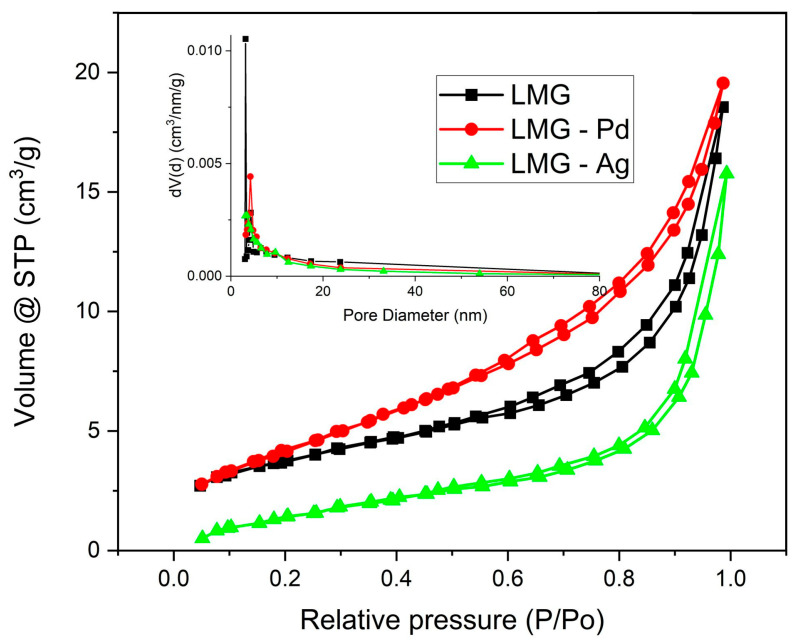
The N_2_ adsorption–desorption isotherms of LMG, LMG-Pd, and LMG-Ag.

The N_2_ adsorption–desorption isotherms of the hybrid materials are presented in Figure 2. The N_2_ adsorption–desorption isotherms of LaMnO_3_, Pd-doped LaMnO_3_, and Ag-doped LaMnO_3_ are type IV isotherms, characteristic of mesoporous materials, with a clear hysteresis loop associated with capillary condensation. This behavior indicates the presence of a well-developed mesoporous structure, typical for perovskite oxides synthesized via the sol–gel method.

Doping with Pd and Ag induces noticeable changes in the textural properties of LaMnO_3_. The doped samples show a higher nitrogen uptake at high relative pressures (P/P_0_ > 0.8) compared to pristine LaMnO_3_, suggesting an increased contribution of mesopores and a possible enhancement of the specific surface area and total pore volume. These textural modifications are expected to be beneficial for catalytic and photocatalytic applications by increasing the number of accessible active sites and improving mass transfer.

Table 2 shows the textural parameters data obtained from the N_2_ isotherms.

The BET surface area values indicate that Pd doping slightly increases the specific surface area of LaMnO_3_ (15.7 m^2^/g) compared to the pristine sample (13.7 m^2^/g), which is beneficial for electrocatalytic applications in alkaline water electrolysis, as a higher surface area generally provides a larger number of accessible active sites. In contrast, Ag-doped LaMnO_3_ exhibits a lower surface area (8.3 m^2^/g), which may limit the density of electrochemically active sites and partially explain differences in electrocatalytic performance.

### 3.3. Water Electrolysis Study

**Figure 3:**
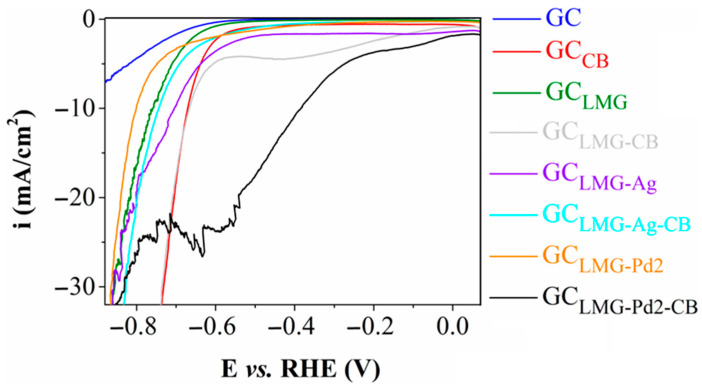
Cathodic polarization curves recorded on the GC, GC_CB_, GC_LMG_, GC_LMG-CB_, GC_LMG-Ag_, GC_LMG-Ag-CB_, GC_LMG-Pd2_, and GC_LMG-Pd2-CB_ electrodes. Electrolyte solution: 1 M KOH. v = 5 mV/s.

The cathodic polarization curves obtained on the modified electrodes are shown in Figure 3. It can be seen that the linear sweep voltammogram recorded on the GC_LMG-Pd2-CB_ electrode stands out by reaching the −10 mA/cm^2^ current density at the lowest electrochemical potential value. The η_HER_ value corresponding to this current density is often determined and used to compare the electrocatalytic activities of different electrocatalysts [31]. Table 3 presents the η_HER_ values found for the studied electrodes.

Since GC_LMG-Pd2-CB_ shows the highest electrocatalytic activity, its electrochemical properties were further studied and compared to those of GC_LMG-Pd2_. The electrical double layer capacitance (C_dl_), R_f_, ECSA, and the Tafel slope were calculated based on the acquired experimental data, and their values are shown in Table 4. All the obtained values evidence the superior electrochemical properties of the GC_LMG-Pd2-CB_ electrode. Importantly, the ECSA value is directly proportional to the number of electrocatalytically active sites involved in an electrocatalytic process [32]. The higher ECSA value of GC_LMG-Pd2-CB_ helps to explain its higher electrocatalytic activity. Electrodes manufactured using carbon black can benefit from an increase in the number of their catalytic centers exposed to the electrolyte solution [33,34].

**Figure 4:**
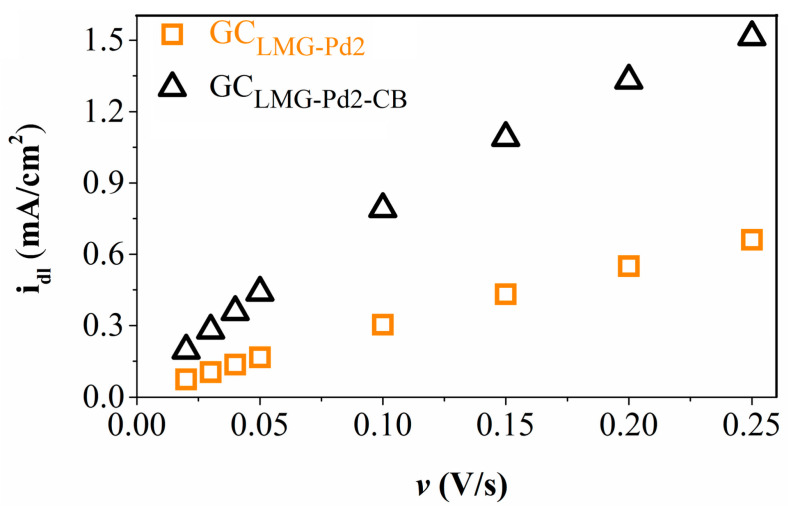
The capacitive current density vs. scan rate plots obtained for the GC_LMG-Pd2_ and the GC_LMG-Pd2-CB_ electrodes.

To obtain the C_dl_ values from Table 4, cyclic voltammograms were recorded at increasing scan rate values. These are shown in Figure S4 from the Supplementary Material file (Figure S4a for GC_LMG-Pd2_ and Figure S4b for GC_LMG-Pd2-CB_). The acquired data were used to calculate the i_dl_ values, subsequently represented in the i_dl_ vs. scan rate plot from Figure 4. The C_dl_ values are the slopes of the curves in Figure 4 and were used to calculate the R_f_ and ECSA parameter values [23].

**Figure 5:**
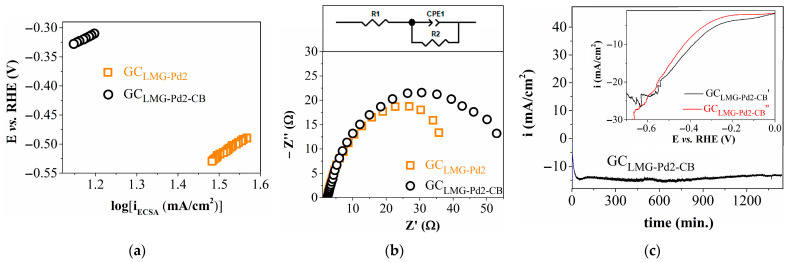
(**a**) HER Tafel plots for the GC_LMG-Pd2_ and GC_LMG-Pd2-CB_ electrodes in 1 M KOH solution. The current density (i_ECSA_) is ECSA-normalized. (**b**) Nyquist plots of GC_LMG-Pd2_ and GC_LMG-Pd2-CB_ obtained in 1 M KOH solution. The inset shows the equivalent circuit. (**c**) Chronoamperometric curve recorded on GC_LMG-Pd2-CB_ in 1 M KOH solution and inset showing the cathodic polarization curves obtained on GC_LMG-Pd2-CB_ before (GC_LMG-Pd2-CB_′) and after (GC_LMG-Pd2-CB_″) the electrochemical stability experiment (1 M KOH solution, scan rate = 5 mV/s).

The catalytic kinetics at the interface between the electrodes and the electrolyte solution were also investigated. The Tafel and Nyquist plots are shown in Figure 5a and Figure 5b, respectively. Figure 5c presents the chronoamperometric curve recorded during the electrochemical stability test performed on GC_LMG-Pd2-CB_.

A low Tafel slope value means faster reaction kinetics [35]. However, even though the value determined for GC_LMG-Pd2-CB_ is lower than the one found for GC_LMG-Pd2_, it is still characteristic of electrocatalysts with sluggish HER kinetics. When the Tafel slope is in the 40 to 120 mV/dec, the HER is probably unfolding through a Volmer–Heyrovsky mechanism, but when the slope value is higher than 120 mV/dec, and the reaction takes place in a strong alkaline environment, the probable mechanism is a Volmer-limited one, with the Heyrovsky step slower than the Volmer step [36,37]. The Tafel slope is affected by the pH of the electrolyte solution, and at high pH values, most of the electrode’s surface becomes covered by hydrogen bubbles, which can cause the slope value to surpass 120 mV/dec [36,38].

The EIS spectra of GC_LMG-Pd2_ and GC_LMG-Pd2-CB_ were recorded at the η_HER_ values corresponding to i = −10 mA/cm^2^. The electrodes were biased at 10 mV within the 10^5^ to 10^−2^ Hz frequency range. The EIS measurements under HER conditions provide the charge transfer resistance (R2) of the two electrodes. A semicircle can be observed in both cases, the size of which is a quantitative indicator of R2 at the electrode/electrolyte interface during the HER [39]. The Nyquist plots were fitted with the Randles equivalent circuit (inset of Figure 5b), using ZView software (Version: 2.80, Scribner Associates Inc., Southern Pines, NC, USA). The simple circuit consists of the Ohmic resistance (R1), the constant phase element (CPE1), and the charge transfer resistance (R2). The small R1 values determined for the two electrodes (2.51 Ω for GC_LMG-Pd2_ and 2.38 Ω for GC_LMG-Pd2-CB_) point to a close contact between the electrocatalyst and the GC support. However, the R2 value obtained for GC_LMG-Pd2_ is smaller than the value found for GC_LMG-Pd2-CB_ (43.68 Ω vs. 58.17 Ω). This suggests that the addition of carbon black did not have the intended effect of enhancing electrical conductivity. Still, it is probably responsible for GC_LMG-Pd2-CB_’s higher ECSA value.

The overall conclusion of the comparative evaluation of GC_LMG-Pd2_ and GC_LMG-Pd2-CB_ regarding their electrochemical properties is that the latter electrode possesses superior characteristics and displays a higher HER water electrolysis activity.

**Figure 6:**
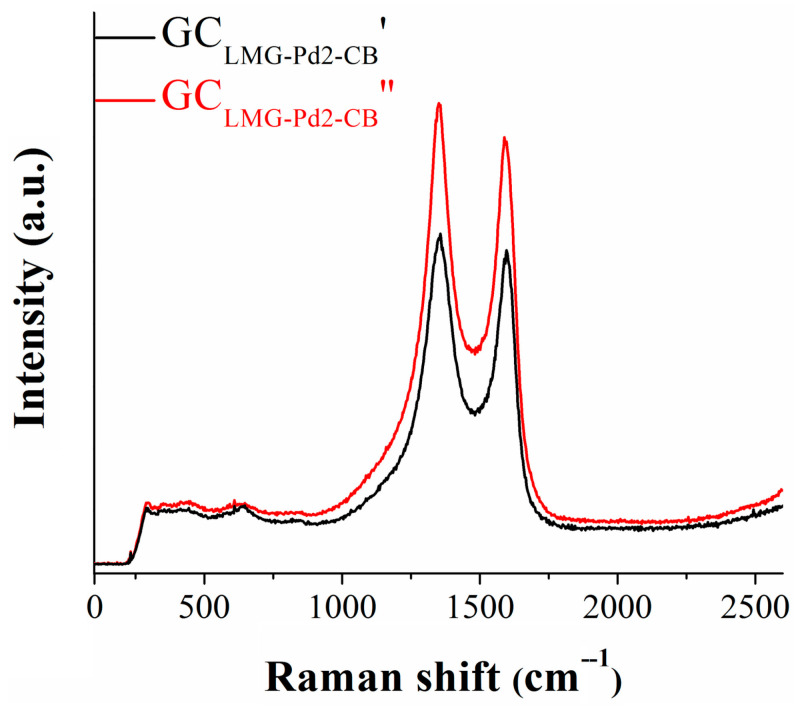
Raman spectra obtained on GC_LMG-Pd2-CB_ before (GC_LMG-Pd2-CB_′) and after (GC_LMG-Pd2-CB_″) the amperometric stability test.

The electrochemical stability of GC_LMG-Pd2-CB_ was investigated during a 24 h chronoamperometric experiment at a constant potential value corresponding to i = −10 mA/cm^2^. After the first hour and until the end of the test, the recorded i-t curve (Figure 5c) shows shifts in current density, between −13 and −16 mA/cm^2^, indicating the relative stability of the studied electrode. The inset of Figure 5c presents the cathodic polarization curves obtained before (GC_LMG-Pd2-CB_′) and after (GC_LMG-Pd2-CB_″) the amperometric experiment. The η_HER_ value at i = −10 mA/cm^2^ increased by ~40 mV following the test. Because this difference is somewhat higher than the differences often observed when performing stability tests relevant to the water splitting domain [40,41,42], Raman analysis was used to verify the occurrence of any changes in the chemical structure of the electrocatalyst. The spectra recorded on the GC_LMG-Pd2-CB_ electrode, before (GC_LMG-Pd2-CB_′) and after (GC_LMG-Pd2-CB_″) the stability test, are given in Figure 6.

The analyzed samples (GC_LMG-Pd2-CB_′ and GC_LMG-Pd2-CB_″) revealed the same structure since there were no visible changes in the 300–650 cm^−1^ specific LaMnO_3_ bands corresponding to the Mn-O bond [43]. However, the aforementioned bands were weak in comparison to the carbon-specific bands located in the 1350–1600 cm^−1^ region. The LaMnO_3_ bands appeared like “noise” in the Raman spectra. After the chronoamperometric experiment, there were no shifts of the carbon-specific bands (1358 cm^−1^ and 1601 cm^−1^), only slight width modifications, suggesting the presence of induced defects [44]. The changes in peak intensities were due to the defects that appeared after the chronoamperometric experiment as a consequence of the reduction of oxygenated surface functional groups. The application of a negative potential led to a decrease in oxygen-containing bonds, particularly C–O (epoxy) and C=O groups. As oxygen is removed, the intensity of sp^3^ C–C bonds increases, effectively restoring the sp^2^ carbon network of the material [45,46].

Lastly, the HER electrocatalytic activity of GC_LMG-Pd2-CB_ was compared to that of other perovskite oxide-based electrodes reported in the scientific literature and also studied in a 1 M KOH electrolyte solution. The results are presented in Table S2 from the Supplementary Material file. They show that the η_HER_ value of 0.385 V is lower than most of the values mentioned for the identified electrodes. Out of the 35 values from published literature studies, 22 are higher than the value found in the present investigation. Furthermore, some of the remaining values are comparable to it. These observations position the GC_LMG-Pd2-CB_ electrode relatively high among perovskite oxide-based electrodes previously investigated regarding their water splitting electrocatalytic activity.

## 4. Conclusions

In this work, a new class of noble metal-doped LaMnO_3_/graphene oxide (GO) hybrid materials was successfully synthesized and evaluated as electrocatalysts for alkaline water electrolysis. Ultrasonic functionalization enabled effective anchoring of GO onto pristine and Ag- or Pd-doped LaMnO_3_ surfaces, while preserving the perovskite crystalline structure, as confirmed by XRD. BET analysis revealed that Pd doping increased the specific surface area from 13.7 m^2^/g for pristine LaMnO_3_ to 15.7 m^2^/g, while Ag doping decreased it to 8.3 m^2^/g. Total pore volume also increased slightly upon Pd doping (0.0303 cm^3^/g) compared to pristine LaMnO_3_ (0.0288 cm^3^/g), indicating improved mesoporosity. AFM measurements showed that Pd-doped LaMnO_3_/GO exhibited the highest surface roughness. Electrochemical tests demonstrated that the GC_LMG-Pd2-CB_ electrode obtained using the Pd-doped LaMnO_3_/GO hybrid in combination with carbon black achieved the lowest HER overpotential of 0.385 V at −10 mA/cm^2^, outperforming the other electrodes. This study represents the first report on LaMnO_3_/GO hybrids, both undoped and noble metal-doped, as electrocatalysts for alkaline water electrolysis. Future work will focus on improving the Tafel slope by employing advanced electrode modification techniques such as pulsed laser deposition (PLD).

## Figures and Tables

**Table 1:** Electrode names and the contents of the catalyst inks.

Electrode Names	LMG (mg)	LMG-Ag (mg)	LMG-Pd (mg)	Carbon Black (mg)	Nafion Solution (µL)	Ethanol (µL)
GC_0_	-	-	-	-	-	-
GC_CB_	-	-	-	5	50	450
GC_LMG_	5	-	-	-	50	450
GC_LMG-CB_	5	-	-	5	100	900
GC_LMG-Ag_	-	5	-	-	50	450
GC_LMG-Ag-CB_	-	5	-	5	100	900
GC_LMG-Pd2_	-	-	5	-	50	450
GC_LMG-Pd2-CB_	-	-	5	5	100	900

**Table 2:** Textural parameters data obtained from the N_2_ isotherms.

Sample Name	Surface Area, BET, (m^2^/g)	BJH, Average Pore Size Distribution from Desorption Branch (nm)	Total Pore Volume (cm^3^/g)
LMG	13.7	3.182	2.877 × 10^−2^ cc/g for pores smaller than 141.9 nm
LMG-Pd	15.7	4.241	3.032 × 10^−2^ cc/g for pores smaller than 148.4 nm
LMG-Ag	8.3	3.482	9.032 × 10^−3^ cc/g for pores smaller than 143.7 nm

**Table 3:** HER overpotential values determined for the studied electrodes at i = −10 mA/cm^2^.

Electrode	GC	GC_CB_	GC_LMG_	GC_LMG-CB_	GC_LMG-Ag_	GC_LMG-Ag-CB_	GC_LMG-Pd2_	GC_LMG-Pd2-CB_
η_HER_ (V)	-	0.670	0.760	0.667	0.704	0.744	0.801	0.385

**Table 4:** The electrochemical parameter values determined for the GC_LMG-Pd2_ and GC_LMG-Pd2-CB_ electrodes.

Parameters	C_dl_ (mF/cm^2^)	R_f_	ECSA (cm^2^)	HER Tafel Slope (V/dec)
Electrodes				
GC_LMG-Pd2_	2.5638 (R^2^ = 0.9976)	42.73	11.964	0.48 (R^2^ = 0.9911)
GC_LMG-Pd2-CB_	5.8615 (R^2^ = 0.9852)	97.692	27.354	0.34 (R^2^ = 0.9985)

## Data Availability

The data will be available upon reasonable request.

## Supplementary Materials

The following supporting information can be downloaded at: https://www.mdpi.com/article/10.3390/nano16020107/s1. Figure S1. 3D AFM image (a) and AFM profile image of selected area (b) obtained for the GC_LMG_ sample; Figure S2. 3D AFM image (a) and AFM profile image of selected area (b) obtained for the GC_LMG-Ag_ sample; Figure S3. 3D AFM image (a) and AFM profile image of selected area (b) obtained for the GC_LMG-Pd2_ sample; Figure S4. (a) Cyclic voltammograms recorded on GC_LMG-Pd2_ in the −250–100 mV range, at increasing scan rate values (20, 30, 40, 50, 100, 150, 200, and 250 mV/s). (b) Cyclic voltammograms recorded on GC_LMG-Pd2-CB_ in the −250–100 mV range, at increasing scan rate values (20, 30, 40, 50, 100, 150, 200, and 250 mV/s). Electrolyte solution: 0.1 M KCl; Table S1. The values of the AFM parameters; Table S2. The HER activity of the GC_LMG-Pd2-CB_ electrode and of other perovskite oxide-based electrodes in 1 M KOH solution. The η_HER_ values are read at i = −10 mA/cm^2^. References [19,38,47,48,49,50,51,52,53,54,55,56,57,58,59,60] are cited in the Supplementary Material file.
