# Supplementary material for: Noble Metal-Doped Perovskite–GO Hybrids as Efficient Electrocatalysts for Alkaline Water Electrolysis
Source: Nanomaterials (Basel). 2026 Jan 14;16(2):107. doi: 10.3390/nano16020107 (PMC12844288; doi:10.3390/nano16020107)
Supplement: Supplementary file 1 [file nanomaterials-16-00107-s001.zip › nanomaterials-4102891-supplementary.pdf]

Supplementary Material

Noble metal-doped perovskite–GO hybrids as efficient electrocatalysts for alkaline water electrolysis

Bogdan-Ovidiu Taranu [1], Paula Svera[1], Doru Buzatu[1], Maria Poienar[2] and Paula Sfirloaga[1,*]

[1]*National Institute for Research and Development in Electrochemistry and Condensed Matter Timisoara (INCEMC), Dr. Aurel Paunescu Podeanu Str. No. 144, 300569, Timisoara, Romania*
[2]*ICAM -Institute for Advanced Environmental Research, West University of Timișoara, Bd. V. Pârvan No.4, 300223 Timișoara, România*

**Correspondence: paulasfirloaga@gmail.com*

Figures

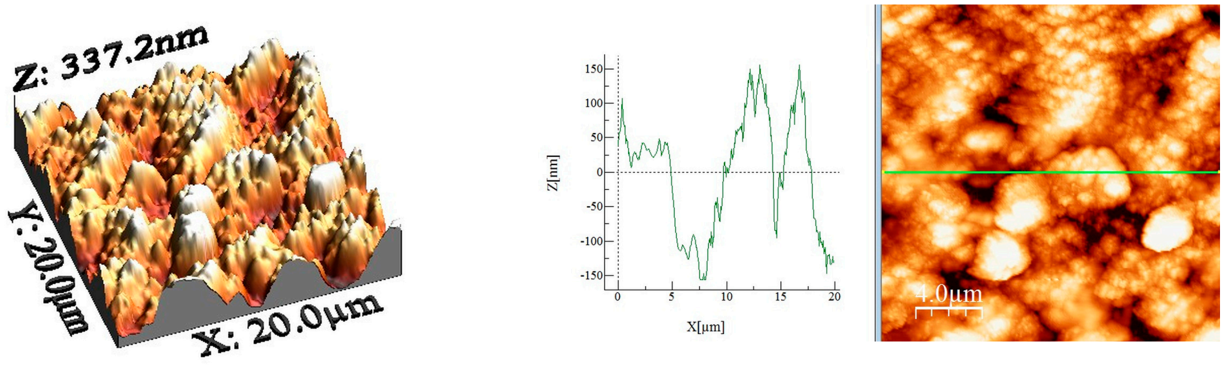

(a) (b)

Figure S1. 3D AFM image (a) and AFM profile image of selected area (b) obtained for the GC_{LMG} sample.

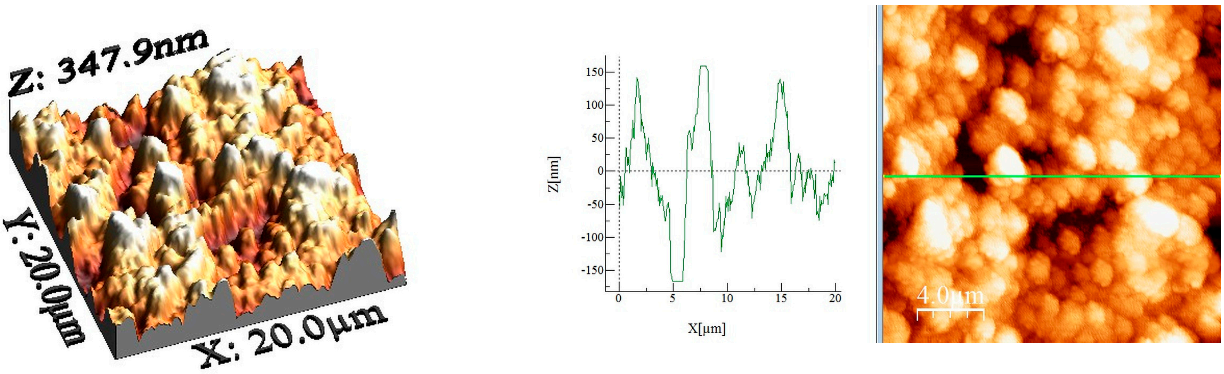

(a) (b)

Figure S2. 3D AFM image (a) and AFM profile image of selected area (b) obtained for the GC_{LMG-Ag} sample.

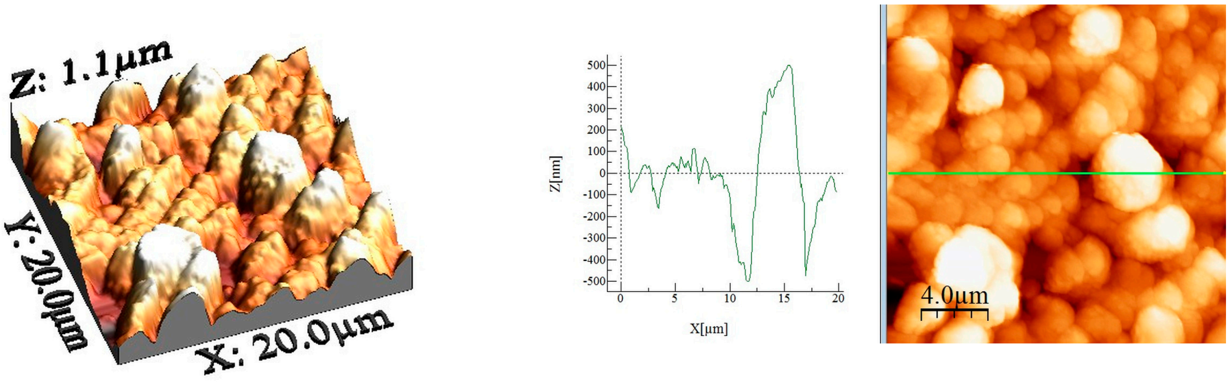

(a) (b)

Figure S3. 3D AFM image (a) and AFM profile image of selected area (b) obtained for the $GC_{LMG-Pd2}$ sample.

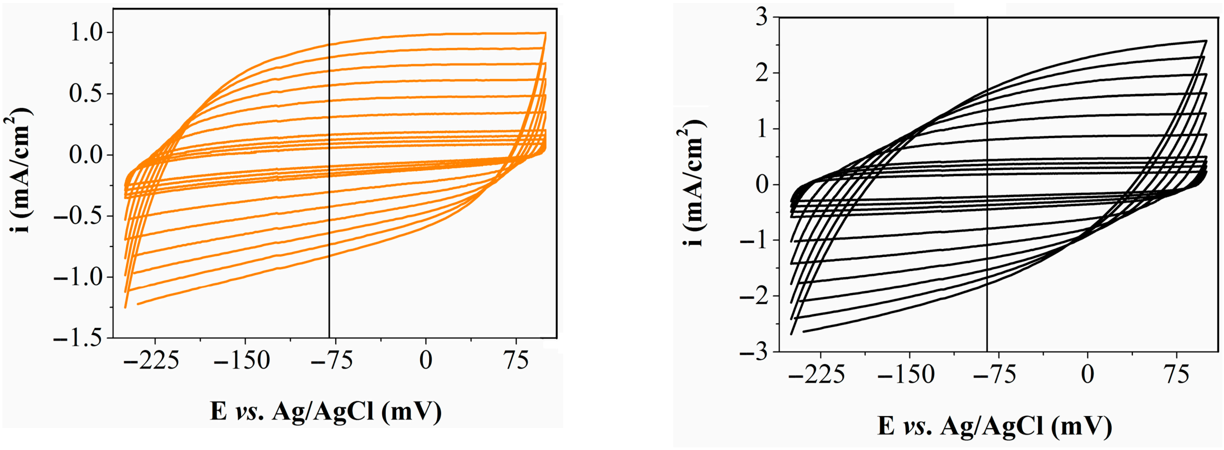

(a) (b)

Figure S4. (a) Cyclic voltammograms recorded on $GC_{LMG-Pd2}$ in the –250 – 100 mV range, at increasing scan rate values (20, 30, 40, 50, 100, 150, 200, and 250 mV/s). (b) Cyclic voltammograms recorded on $GC_{LMG-Pd2-CB}$ in the –250 – 100 mV range, at increasing scan rate values (20, 30, 40, 50, 100, 150, 200, and 250 mV/s).
Electrolyte solution: 0.1 M KCl.

Tables

Table S1. The values of the AFM parameters

Sample name	Ironed area (μm²)	Sa (nm)	Sq (nm)	Sp (nm)	Sv (nm)	Sy (nm)	Sku	Ssk
GC_{LMG}	416.21	61.36	75.01	171.72	–165.50	337.22	2.427	–0.004
$GC_{LMG\text{-}Ag}$	420.42	62.82	77.77	171.29	–176.58	347.87	2.525	–0.106
$GC_{LMG\text{-}Pd2}$	449.04	184.2	233.6	550.4	–574	1124.4	2.759	0.287

Table S2 shows a comparison between the HER electrocatalytic activity of the $GC_{LMG\text{-}Pd2\text{-}CB}$ electrode and other perovskite oxide-based electrodes reported in the scientific literature [S1-S5].

Table S2. The HER activity of the $GC_{LMG\text{-}Pd2\text{-}CB}$ electrode and of other perovskite oxide-based electrodes in 1 M KOH solution. The η_{HER} values are read at i = –10 mA/cm²

Electrocatalyst	η_{HER} (mV)	Ref.
Pd_2-doped $LaMnO_3$/GO + Carbon Black	385	This work
Pd_2-substituted $LaMnO_3$ + Carbon Black	490	[S2]
$BaNiO_3$	427	[S3]
$SrTiFe_{0.9}Pr_{0.1}O_{6-\delta}$	182.4	[S4]
$LaMnO_3$ functionalized montmorillonite	480	[S5]
$LaBaCo_2O_{5+\delta}$	156	
$NdBaCo_2O_{5+\delta}$	363	
$SmBaCo_2O_{5+\delta}$	400	[S6]
$GdBaCo_2O_{5+\delta}$	401	
$EuBaCo_2O_{5+\delta}$	441	
$LaCa_2Fe_3O_8$	400	[S7]
$LaSr_2Fe_3O_8$	~775	
$SrLaCoO_{4-\delta}$	541	
$SrLaCo_{0.5}Fe_{0.5}O_4$	622	[S8]
$LaFeO_4$	691	
$La_{0.96}Ce_{0.04}CoO_{3-\delta}$	305	
$La_{0.98}Ce_{0.02}CoO_{3-\delta}$	373	
$La_{0.94}Ce_{0.06}CoO_{3-\delta}$	413	[S9]
$La_{0.92}Ce_{0.08}CoO_{3-\delta}$	448	
$La_{0.90}Ce_{0.10}CoO_{3-\delta}$	475	
$LaCoO_3$	494	
$BaMoO_3$	336	[S10]
$BaMoO_4$	561	
$CaSrFeMnO_{6-\delta}$	390	[S11]
Core–shell $Co(OH)_2/Sr_2Fe_{1.5}Mo_{0.5}O_{6-\delta}$–NF hybrid	312	
$Sr_2Fe_{1.5}Mo_{0.5}O_{6-\delta}$–NF	370	[S12]
$Sr_2Fe_{1.5}Mo_{0.5}O_{6-\delta}$–bulk	443	
$LaFe_{0.8}Co_{0.2}O_3$	440	[S13]
$LaFe_{0.8}Co_{0.2}O_3$ reduced in H_2	400	
$Ba_{0.95}Co_{0.4}Fe_{0.4}Zr_{0.1}Y_{0.1}O_{3-\delta}$	360	[S14]
$BaCo_{0.4}Fe_{0.4}Zr_{0.1}Y_{0.1}O_{3-\delta}$	~390	
$SrCo_{0.7}Fe_{0.25}Mo_{0.05}O_{3-d}$	323	
$SrCo_{0.7}Fe_{0.2}Mo_{0.1}O_{3-d}$	353	[S15]
$SrCo_{0.7}Fe_{0.3}O_{3-d}$	378	
$La_{0.5}Sr_{0.5}CoO_{3-\delta}$/ $MoSe_2$/ Carbon Black	~240	[S16]
$La_{0.5}Sr_{0.5}CoO_{3-\delta}$	~425	

References

[S1] S. Alom, C.C.W. Kananke-Gamage, F. Ramezanipour, Perovskite oxides as electrocatalysts for hydrogen evolution reaction, *ACS Omega* 7 (2022) 7444–7451, https://doi.org/10.1021/acsomega.1c07203.

[S2] P. Sfirloaga, B.-O. Taranu, M. Poienar, P. Vlazan, Addressing electrocatalytic activity of metal-substituted lanthanum manganite for the hydrogen evolution reaction, *Surfaces and Interfaces* 39 (2023) 102881, https://doi.org/10.1016/j.surfin.2023.102881.

[S3] J. Junita, D. Jayalakshmi, J.D. Rodney, Combustion-derived $BaNiO_3$ nanoparticles as a potential bifunctional electrocatalyst for overall water splitting, *International Journal of Hydrogen Energy* 48 (2023) 14287-14298, https://doi.org/10.1016/j.ijhydene.2022.12.291.

[S4] Q. Sarmad, U.M. Khan, M.M. Baig, M. Hassan, F.A. Butt, A.H. Khoja, R. Liaquat, Z.S. Khan, M. Anwar, S.A. Muhammed Ali, Praseodymium-doped $Sr_2TiFeO_{6-\delta}$ double perovskite as a bi-functional electrocatalyst for hydrogen production through water splitting, *Journal of Environmental Chemical Engineering* 10 (2022) 107609, https://doi.org/10.1016/j.jece.2022.107609.

[S5] B.-O. Taranu, P. Vlazan, P. Svera (m. Ianasi), M. Poienar, P. Sfirloaga, New functional hybrid materials based on clay minerals for enhanced electrocatalytic activity, *Journal of Alloys and Compounds* 892 (2021) 162239, https://doi.org/10.1016/j.jallcom.2021.162239.

[S6] Y. Dou, Y. Xie, X. Hao, T. Xia, Q. Li, J. Wang, L. Huo, H. Zhao, Addressing electrocatalytic activity and stability of $LnBaCo_2O_{5+\delta}$ perovskites for hydrogen evolution reaction by structural and electronic features, *Applied Catalysis B: Environmental* 297 (2021) 120403, https://doi.org/10.1016/j.apcatb.2021.120403.

[S7] S.B. Karki, A.N. Andriotis, M. Menon, F. Ramezanipour, Bifunctional water-splitting electrocatalysis achieved by defect order in $LaA_2Fe_3O_8$ (A = Ca, Sr), *ACS Appl. Energy Mater* 4 (2021) 12063-12066.

[S8] M.S. Alom, F. Ramezanipour, Layered oxides $SrLaFe_{1-x}Co_xO_{4-\delta}$ (x=0–1) as bifunctional electrocatalysts for water-splitting, *ChemCatChem* 13 (2021) 3510-3516, https://doi.org/10.1021/acsaem.1c02028.

[S9] D. Ji, C. Liu, Y. Yao, L. Luo, W. Wang, Z. Chen, Cerium substitution in $LaCoO_3$ perovskite oxide as bifunctional electrocatalysts for hydrogen and oxygen evolution reactions, *Nanoscale* 13 (2021) 9952-9959, https://doi.org/10.1039/D1NR00069A.

[S10] X. Xu, Y. Pan, Y. Zhong, L. Ge, S.P. Jiang, Z. Shao, From scheelite $BaMoO_4$ to perovskite $BaMoO_3$: Enhanced electrocatalysis toward the hydrogen evolution in alkaline media, Composites Part B: Engineering 198 (2020) 108214, https://doi.org/10.1016/j.compositesb.2020.108214.

[S11] R.K. Hona, S.B. Karki, F. Ramezanipour, Oxide electrocatalysts based on earth-abundant metals for both hydrogen- and oxygen-evolution reactions, *ACS Sustainable Chemistry & Engineering Journal* 8 (2020) 11549-11557, https://doi.org/10.1021/acssuschemeng.0c02498.

[S12] B. He, K. Tan, Y. Gong, R. Wang, H. Wang, L. Zhao, Coupling amorphous cobalt hydroxide nanoflakes on $Sr_2Fe_{1.5}Mo_{0.5}O_{5+\delta}$ perovskite nanofibers to induce bifunctionality for water splitting, *Nanoscale* 12 (2020) 9048-9057, https://doi.org/10.1039/D0NR00848F.

[S13] L. Tang, Z. Chen, F. Zuo, B. Hua, H. Zhou, M. Li, J. Li, Sun, Y., Enhancing perovskite electrocatalysis through synergistic functionalization of B-site cation for efficient water splitting, *Chemical Engineering Journal* 401 (2020) 126082, https://doi.org/10.1016/j.cej.2020.126082.

[S14] Q.A. Islam, R. Majee, S. Bhattacharyya, Bimetallic nanoparticle decorated perovskite oxide for state-of-the-art trifunctional electrocatalysis, *Journal of Materials Chemistry A* 7 (2019) 19453-19464, https://doi.org/10.1039/C9TA06123A.

[S15] Z. Zhang, Y. Chen, Z. Dai, S. Tan, D. Chen, Promoting hydrogen-evolution activity and stability of perovskite oxides via effectively lattice doping of molybdenum, *Electrochimica Acta* 312 (2019) 128-136, https://doi.org/10.1016/j.electacta.2019.04.163.

[S16] N.K.Oh, C. Kim, J. Lee, O. Kwon, Y. Choi, G.Y. Jung, H.Y. Lim, S.K. Kwak, G. Kim, H. Park, In-situ local phase-transitioned $MoSe_2$ in $La_{0.5}Sr_{0.5}CoO_{3-\delta}$ heterostructure and stable overall water electrolysis over 1000 hours, Nature Communications 10 (2019) 1723, https://doi.org/10.1038/s41467-019-09339-y.
